# Supplementary figures and images for: Polyurethane Foam Residue Biodegradation through the Tenebrio molitor Digestive Tract: Microbial Communities and Enzymatic Activity
Source: Polymers (Basel). 2022 Dec 31;15(1):204. doi: 10.3390/polym15010204 (PMC9823465; doi:10.3390/polym15010204)

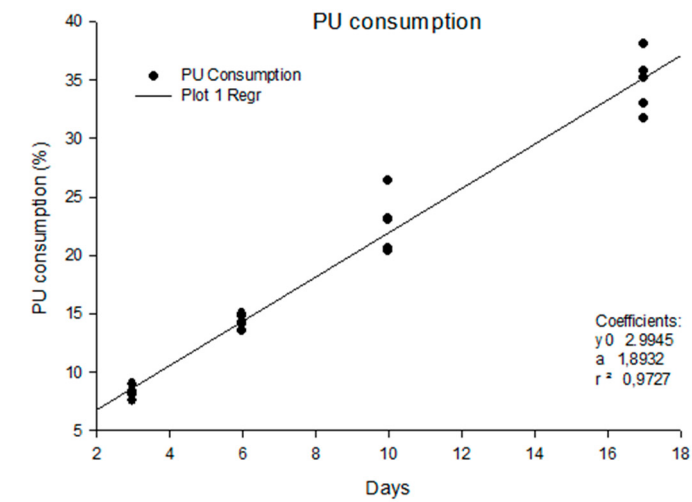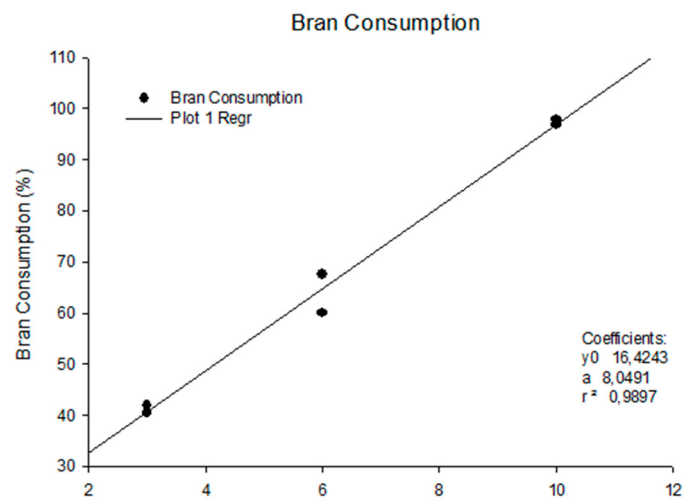

Figure S1. Spearman Correlation between PU and Bran consumption and days of the experiment.

Supplement: Supplementary file 1 [file polymers-15-00204-s001.zip › polymers-2083254-supplementary.pdf]
